# Slowly developing toxic epidermal necrolysis-like reaction associated with pemetrexed and carboplatin

**DOI:** 10.3332/ecancer.2020.1010

**Published:** 2020-02-13

**Authors:** Gerhard Eichhoff

**Affiliations:** Capital and Coast District Health Board, Dermatology Service, Wellington Hospital, Wellington, New Zealand

**Keywords:** toxic epidermal necrolysis, cutaneous adverse drug reactions, drug rash, pemetrexed

## Abstract

Most cutaneous adverse drug reactions reported in association with chemotherapy, such as a limited maculopapular rash, are considered mild and do not affect the continuation of the treatment. Toxic epidermal necrolysis (TEN), however, is a life-threatening reaction that needs treatment discontinuation. The present case shows the slow progression from a pemetrexed and carboplatin-associated maculopapular rash to a TEN-like reaction.

## Introduction

Toxic epidermal necrolysis is a serious adverse drug reaction (sADR) linked to high morbidity and mortality. The usual course of toxic epidermal necrolysis (TEN) is a rapid progression from an erythema to epidermolysis [1]. The multitargeted antifolate pemetrexed is frequently associated with maculopapular rash and is a known elicitor for TEN, whereas the combination with carbo or cisplatin does not substantially increase the risk for cutaneous adverse events [2, 3].

## Clinical case

**Figure 1.**
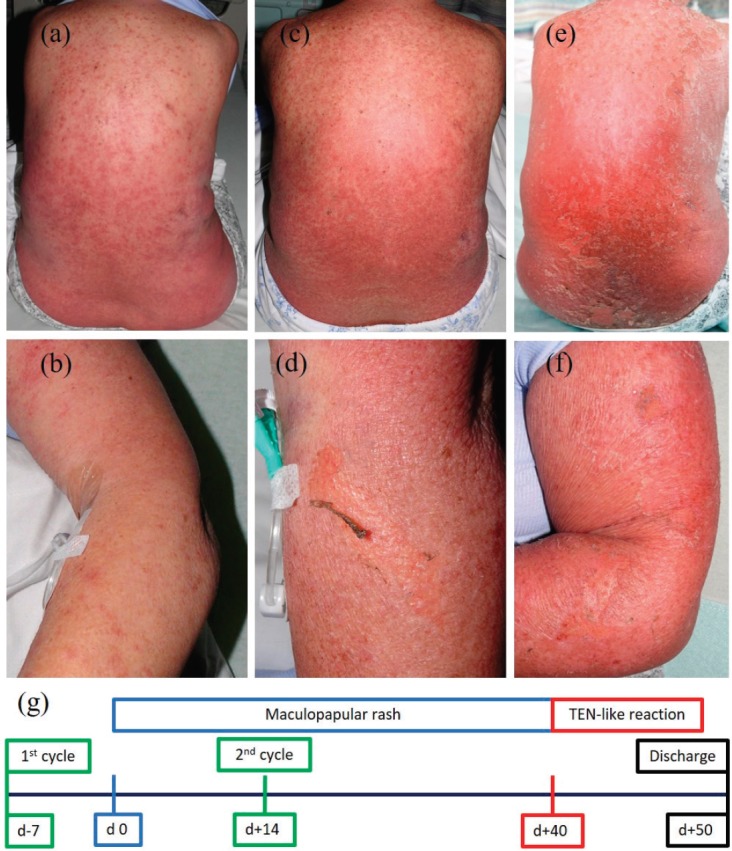
Development of the rash and timeline. Maculopapular eruptions on back (a) and left forearm (b) 27 days after onset of the rash. Coalescing and spreading maculopapular eruptions on back (c) and left forearm (d) together with beginning denudation 40 days after onset of the rash. Progression to widespread denudation on back (e) and left forearm (f) over the next days. Schematic timeline of events (g) with onset of the rash on day 0 (d 0).

A 70-year-old female patient without other relevant medical history was started on pemetrexed and carboplatin for mesothelioma after vitamin B12 and folic acid supplementation. The patient developed a rash on the anterior chest wall 7 days after the first cycle which persisted despite treatment with loratadine. Four days after the second cycle (14 days after the onset of the rash), the patient was admitted with widespread maculopapular eruptions treated with betamethasone valerate 0.1% cream along with cetirizine 10 mg twice daily. The patient’s additional symptoms were fever, pancytopenia, including neutropenia, seizures and confusion, pulmonary embolism, and nausea. Over the next few days, the rash continued to coalesce and spread. Forty days after the initial onset, 80% of the total body’s surface area (TBSA) was affected with beginning denudation (Figure 1). Histology showed widespread epidermal necrosis with multiple dyskeratotic keratinocytes. A TEN-like reaction was diagnosed. Treatment with prednisone 50 mg daily to be weaned off by 10 mg every 7 days was initiated along with supportive measures. The patient could be discharged to the community in a stable condition 50 days after the initial rash. Chemotherapy was discontinued and palliative care was initiated due to the sADRs including the diagnosis of a TEN-like reaction.

## Discussion

Cutaneous ADR, usually grade 1– and grade 2 rashes are commonly seen with pemetrexed use, whereas Piérard-Franchemont *et al* [2] highlighted the difficulties interpreting these reactions as they are frequently labelled using the unspecific term ‘skin rash’.

It is debated whether pemetrexed exhibits a direct cytotoxic effect of on the keratinocytes or elicits an indirect immune response underlying the pathophysiology of the drug’s associated cutaneous toxicity [2, 4]. The so-called antifolate cytotoxic skin reactions can clinically resemble TEN [4].

Supplementation of folic acid and vitamin B12 is routinely provided 1 week prior to chemotherapy aiming to minimise its cytotoxic effects, whereas the administration of oral or intravenous dexamethasone does not seem to reduce pemetrexed-associated cutaneous ADRs [4, 5].

To the author’s knowledge, five cases of TEN-like reactions associated with pemetrexed were described in the literature to date. Tummino *et al* [6] reported on the appearance of a TEN-like reaction 15 days after administration of the second cycle of pemetrexed given for a refractory non-mall cell lung cancer. Another case of a TEN-like reaction including mucosal lesions and haemorrhagic blisters was noticed after the second cycle of pemetrexed and cisplatin given for metastatic non-small cell lung cancer [7]. Bosch-Barrera *et al* [3] described a case of a TEN-like reaction after the second cycle of pemetrexed, given in conjunction with carboplatin and Vitamin B12 and folic acid supplementation. A case of pemetrexed and carboplatin-associated TEN-like reaction in a patient with non-small cell lung cancer and pre-existing Sharp syndrome occurring primarily in the previous radiation field was reported by Then *et al* [8]. Another case of a TEN-like reaction in association with pemetrexed and cisplatin, given in combination with gefitinib, manifesting after the first cycle was described by Huang *et al* [9]. All of these cases described a rapid progression of the TEN-like reaction and none of the reported TEN-like reactions had a fatal outcome.

Whether or not a chemotherapy-associated adverse drug reaction needs the discontinuation of the treatment depends on the severity of the reaction. Maculopapular eruptions < 30% TBSA are considered grade 1 or 2 adverse events and do not usually lead to discontinuation of treatment. However, TEN-like reactions are regarded as grade 3 or 4 adverse events and require discontinuation [10, 11].

The patient discussed in this study developed a limited maculopapular eruption 7 days after the first cycle of chemotherapy. Therefore, the treatment was continued. After the second cycle, the rash progressed to a grade 4 event with large areas of denudation. The patient’s other chemotherapy-associated sADRs required multiple other medications. The onset of the rash before initiation of these medications argues for a pemetrexed- and carboplatin-associated event. However, the development of a second adverse reaction to one of these medications leading to the TEN-like reaction cannot be completely excluded. A Naranjo score of 6 was calculated, suggesting that the progressing maculopapular eruption was secondary to pemetrexed and carboplatin exposure [12].

## Conclusions

The presented case of a slowly developing TEN-like reaction case aims to raise awareness about the finding that a maculopapular rash associated with pemetrexed and carboplatin can possibly progress into a more serious ADR. This should be taken into consideration when deciding on the continuation of the provoking treatment.

## Conflicts of interest

The author declares that he has no conflicts of interest.

## Funding statement

The author did not receive funding for publication of this manuscript.
